# Risk of Second Primary Cancers in Melanoma Survivors: A Retrospective Hospital-Based Cohort Study from Two Romanian Referral Centres

**DOI:** 10.3390/medsci14040494

**Published:** 2026-08-19

**Authors:** Salomea-Ruth Halmágyi, Loredana Ungureanu, Alina Florentina Vasilovici, Mihail-Alexandru Badea, Ioana-Irina Trufin, Adina Patricia Apostu, Adrian Ilie Pascu, Simona Corina Şenila

**Affiliations:** 1 Department of Dermatology, “Iuliu Hațieganu” University of Medicine and Pharmacy, 400006 Cluj-Napoca, Romania; h_salomea@yahoo.com (S.-R.H.); letca.alina@umfcluj.ro (A.F.V.); ioana.irina.trufin@gmail.com (I.-I.T.); adinna.apostu@yahoo.com (A.P.A.); adr.pascu.97@gmail.com (A.I.P.); corina.senila@umfcluj.ro (S.C.Ş.); 2 Department of Dermatology, Emergency County Hospital, 400006 Cluj-Napoca, Romania; 3 Dermatology Department, The George Emil Palade University of Medicine, Pharmacy, Science, and Technology, 540139 Targu Mures, Romania; mihail.badea@umfst.ro

**Keywords:** melanoma, neoplasms, second primary, cohort studies, incidence, risk factors

## Abstract

Background and objectives: Melanoma survivors may develop additional primary malignancies, but data from Eastern European hospital cohorts are scarce. We aimed to estimate the observed incidence of second primary cancers (SPCs) within a Romanian referral-centre cohort, identify associated factors, and explore the association of SPC occurrence with overall survival. Methods: We conducted a retrospective, hospital-based cohort study of 394 patients with histopathologically confirmed cutaneous melanoma followed at two referral centres in Cluj-Napoca. Additional malignancies were counted as new primaries only when clinical and/or histopathological documentation distinguished them from recurrence or metastasis. Incidence density was expressed per 1000 person-years. Multivariable logistic regression assessed age, sex, residence, and Breslow thickness. Fixed-covariate Cox and Kaplan–Meier analyses were considered exploratory because of immortal-time and competing-risk limitations. Results: Over 1848 person-years, 79 patients (20.1%) developed at least one SPC (131 events; observed incidence 70.9/1000 person-years), most frequently cutaneous (53.6/1000 person-years). SPC occurrence increased with age (Cochran–Armitage Z = 5.63, *p* < 0.001); the Kaplan–Meier complement estimate reached 20.7% at five years. Age independently predicted SPC (OR 1.86 per decade, *p* < 0.001), whereas greater Breslow thickness was inversely associated (OR 0.86, *p* = 0.008). Actinic keratosis showed a strong unadjusted association (OR 6.64, *p* < 0.001) but was not included in the adjusted model. The fixed-status Cox model showed lower mortality among patients with an SPC (HR 0.36, *p* < 0.001), a finding vulnerable to detection and immortal-time bias. Conclusions: SPCs were frequent in this hospital cohort and predominantly cutaneous. Older age was an independent predictor, while actinic keratosis was a strong unadjusted marker. Prospective, population-based validation is needed before surveillance intensity is individualised.

## 1. Introduction

Cutaneous melanoma is among the most rapidly increasing malignancies in fair-skinned populations, and global burden analyses document a steady rise in age-standardised incidence over the past three decades [1]. Improvements in public awareness and the implementation of screening programmes have shifted the diagnostic distribution toward thinner, earlier-stage tumours, while declining mortality reflects both earlier detection and the advent of effective systemic therapy [2]. At a population level, melanoma now contributes a substantial and growing share of cancer-related disability worldwide [3]. Together, these trends mean that the prevalence of melanoma survivors far exceeds the annual number of new diagnoses, and this expanding survivor population raises questions that extend well beyond the index tumour.

A consistent body of evidence indicates that a personal history of melanoma is associated with subsequent primary malignancies. A comprehensive meta-analysis of more than 350,000 patients reported an overall relative risk of roughly 1.6 for non-melanoma second primaries, with the association persisting for decades [4]. Second primary melanomas and non-cutaneous SPCs are clinically distinct. Additional melanomas share many of the same ultraviolet, phenotypic, and inherited susceptibility pathways as the index melanoma and primarily reinforce the need for lifelong dermatologic surveillance [5]. In contrast, non-cutaneous SPCs comprise heterogeneous solid-organ and haematologic malignancies with different latency patterns, treatment implications, and effects on mortality [6,7]. Registry studies have confirmed that the risk of a second melanoma remains elevated long after the first diagnosis, while breast, prostate, and lymphoid malignancies are among the non-cutaneous cancers repeatedly reported in melanoma survivors [5,6].

Recent population-based cohorts have refined these estimates and identified clinical correlates of risk. A Spanish series found that older age, head-and-neck melanoma location, and the lentigo maligna subtype were associated with higher second-primary risk [8], and a nationwide Korean study reported an overall standardised incidence ratio of about 1.5, with comorbidity and elevated body-mass index emerging as associated factors [9]. Keratinocyte carcinomas dominate the cutaneous burden: a meta-analysis estimated several-fold increases in both basal-cell and squamous-cell carcinoma among melanoma survivors [10]. Even survivors of in situ melanoma carry a measurable, if smaller, increase in risk [11]. However, reported associations are not necessarily transportable across populations because constitutional pigmentation and ancestry, patterns of occupational and recreational ultraviolet exposure, screening intensity, access to specialty care, and cancer-registry completeness can all influence which SPCs are detected and how incidence is measured.

Several mechanisms plausibly underlie these associations. Repeated ultraviolet exposure produces cumulative mutational injury across chronically exposed skin, creating fields in which multiple independent keratinocyte cancers and melanomas can arise; this field-cancerization process also varies by melanoma subtype and anatomical pattern of sun exposure [12]. Constitutional susceptibility may act in parallel: a high naevus burden and inherited predisposition can increase the probability of multiple primary melanomas, and such host factors may be particularly relevant among patients who develop more than one melanoma [13]. Immune dysregulation provides a further pathway because impaired tumour immunosurveillance, whether related to haematologic disease or iatrogenic immunosuppression, can facilitate the emergence of additional malignancies [14,15]. These mechanisms are not mutually exclusive and may interact with age, accumulated exposure, and treatment history over long periods of survivorship.

The clinical importance of second primary cancers is not merely epidemiological. The emergence of a second malignancy frequently exerts an independent and adverse effect on overall survival, and the magnitude of that effect depends on the anatomical site and biological aggressiveness of the second tumour. Recognising which survivors are at greatest risk, and over what time horizon, is therefore central to the design of rational long-term follow-up rather than uniform, resource-intensive surveillance of every patient.

Despite the maturity of this literature in Western European, North American, and Australasian registries, comparable data from Eastern Europe, and from Romania in particular, are scarce. Romania may differ from populations represented in large registries with respect to intermittent and occupational ultraviolet exposure, access to dermatologic screening, referral pathways to tertiary centres, and completeness of longitudinal cancer ascertainment. These features can influence both detection and measured SPC incidence. For that reason, estimates from other European populations are useful contextual comparators but were not assumed to be directly interchangeable with a Romanian hospital cohort. Against this background, the present study estimates overall and site-specific SPC incidence within two Romanian referral centres, characterises melanoma-to-SPC latency, evaluates associated clinical factors, and explores the association between SPC status and overall survival. Its principal contribution is to provide the first local hospital-based description of SPC patterns while explicitly identifying the methodological limitations that must be addressed before population-level risk or surveillance recommendations can be established.

## 2. Materials and Methods

### 2.1. Study Design and Population

This was a retrospective, analytic, observational hospital-based cohort study based on the review of existing clinical records of patients with cutaneous melanoma followed in the Department of Dermatology of the Cluj-Napoca County Emergency Clinical Hospital and the “Prof. Dr. Ion Chiricuță” Oncology Institute, Cluj-Napoca. Eligible participants had a histopathologically confirmed primary cutaneous melanoma. No lower or upper age limit was applied at eligibility: all patients with a histopathologically confirmed primary cutaneous melanoma managed at the participating centres were eligible, irrespective of age at diagnosis. The cohort therefore includes one patient who was an adolescent (16.3 years) at the time of melanoma diagnosis, consistent with the age range reported in Table 1. Patients with metastatic melanoma of unidentified primary origin and those without histopathological confirmation of the primary tumour were excluded. The final analytic dataset comprised 394 patients. Because both institutions are tertiary referral centres, the cohort is not population-based and is susceptible to referral, selection, and surveillance bias.

The retrospective cohort included both living patients who provided informed consent and deceased patients identified through institutional records, in order to avoid the survival bias that would arise from restricting the analysis to survivors only. Data were retrospectively extracted from outpatient and inpatient charts and hospital information systems and supplemented, when available, by information obtained through direct or telemedicine-based patient contact. Follow-up extended from the first melanoma diagnosis to death or the last documented clinical contact; patients without a subsequent encounter were censored at their last recorded contact rather than assumed to remain event-free. For analyses of time to first SPC, follow-up ended at the earliest of first SPC diagnosis, death, or last contact. For descriptive incidence densities that counted multiple SPC events, total observed follow-up through death or last contact was used so that recurrent independent primaries detected during continued surveillance were retained.

### 2.2. Variables and Definitions

For every patient we recorded demographic characteristics (age at first melanoma, sex, urban or rural residence), constitutional melanoma risk factors (Fitzpatrick phototype, hair and eye colour, cumulative sun exposure, childhood sunburns, sunscreen use, atypical naevi, high naevus count, solar lentigines, actinic keratosis, and immunosuppression), and general cancer risk factors (smoking expressed in pack-years, alcohol use, occupational exposure to noxious agents, and family history of cancer). Actinic keratosis and related field-change were defined according to current European interdisciplinary criteria [16], and immunosuppression encompassed iatrogenic immunosuppressive therapy, transplantation, haematologic malignancy, and HIV, categories established as cutaneous-cancer risk states [15].

Melanoma was characterised by clinicopathological subtype, anatomical location, Breslow thickness, mitotic rate, ulceration, tumour-infiltrating lymphocytes, sentinel-node status, and TNM stage assigned according to the American Joint Committee on Cancer eighth-edition criteria [17]. Every additional malignancy was documented with its diagnosis date, broad category (cutaneous, solid-organ, haematologic, endocrine, or sarcoma), specific type, stage, and treatment received, including any immunosuppressive or transplant-related exposure relevant to subsequent malignancy [18]. An additional malignancy was counted as a true new primary only when the clinical and/or histopathological record documented an independent tumour. Local recurrence, in-transit disease, nodal or distant melanoma metastasis, and metastatic disease attributed to a known prior cancer were not counted as SPCs. For additional melanomas, a distinct lesion documented as a new primary rather than recurrence or metastasis was required. Malignancies diagnosed before the first melanoma were designated prior cancers, whereas independent primaries diagnosed on or after the melanoma diagnosis date were designated SPCs. Independent cancers detected during the melanoma work-up were therefore retained as synchronous SPCs; the present analysis did not otherwise formally subdivide SPCs into synchronous and metachronous categories. Patients could contribute to both the prior-cancer and SPC categories.

### 2.3. Statistical Analysis

Continuous variables were summarised as mean ± standard deviation and compared with the Welch (unequal-variance) *t*-test; the Kruskal–Wallis test was used for skewed latency distributions. Categorical variables were expressed as counts and percentages and compared with the Fisher exact test for 2 × 2 tables and the Pearson chi-square test for larger tables. Descriptive incidence density for all recorded SPC events, including multiple independent primaries in the same patient, was calculated as the number of SPC events divided by total observed person-years through death or last contact and expressed per 1000 person-years with exact Poisson 95% confidence intervals [19]. Analyses of first-SPC occurrence used the first qualifying SPC as the event. The dose–response relationship between age stratum and SPC occurrence was tested with the Cochran–Armitage trend test [20].

To identify independent predictors of SPC, a complete-case multivariable logistic-regression model was fitted with age (per decade), sex, residence, and Breslow thickness as covariates, with odds ratios and 95% confidence intervals reported. Actinic keratosis was evaluated in unadjusted analyses but was not included in the adjusted model. For descriptive time-to-first-SPC analysis, the cumulative probability of SPC was estimated as the complement of the Kaplan–Meier survival function [21]. Because death was treated as censoring rather than as a competing event, these values should be interpreted as exploratory Kaplan–Meier complement estimates and may overestimate absolute cumulative incidence, particularly at later follow-up. Overall survival was explored with Kaplan–Meier curves, the log-rank test, and a Cox proportional-hazards model adjusted for age, sex, and Breslow thickness [22]. SPC status was treated as a fixed covariate; therefore, the survival association is vulnerable to immortal-time bias and is not a bias-adjusted causal effect estimate [23]. No time-varying SPC model or formal competing-risk model was performed in the current analysis. A two-sided *p*-value below 0.05 was considered statistically significant. Analyses were performed in Python 3.12 using SciPy, statsmodels, and lifelines.

### 2.4. Ethical Considerations and Data Handling

Data generated during routine clinical care were retrospectively reviewed and consolidated in a structured, de-identified electronic database in which patients were represented only by sequential study identifiers. Living participants provided informed consent for inclusion; for deceased patients, data were drawn from existing medical records in accordance with institutional governance. For the single participant who was a minor at the time of melanoma diagnosis, consent and data-handling procedures followed the institutional requirements approved by the ethics committee. The study was conducted in accordance with the Declaration of Helsinki and was approved by the Ethics Committee of the “Prof. Dr. Ion Chiricuță” Oncology Institute, Cluj-Napoca (evaluation report/approval no. 309 of 31 January 2025; application no. 970 of 28 January 2025), within the doctoral research protocol entitled “Asocieri neoplazice în dermatologie”.

To preserve analytic integrity, every derived variable was traced back to its source field, and the temporal classification of malignancies was verified against recorded diagnosis dates. Person-time with implausible negative values arising from data-entry artefacts was truncated at zero for survival modelling, and malignancies with missing diagnosis dates were excluded from the prior-versus-secondary temporal classification rather than arbitrarily assigned. The multivariable models used complete cases and therefore included 381 of 394 patients, excluding 13 patients (3.3%) with incomplete model covariates. The aggregate analytic file available for this study did not retain a separate screening log with the total number and characteristics of records excluded before construction of the final cohort, nor did it retain a count that would permit a formal sensitivity analysis of malignancies excluded because of missing diagnosis dates. These quantities therefore cannot be reconstructed reliably without returning to the source records and are acknowledged as a limitation rather than estimated post hoc.

## 3. Results

The cohort comprised 394 patients with histopathologically confirmed cutaneous melanoma, contributing 1848 person-years of observation. The mean age at first melanoma diagnosis was 55.8 ± 14.7 years, spanning 16.3 to 91.4 years; because no age limit was applied at eligibility (Section 2.1), the cohort included one adolescent at diagnosis, and the study population is therefore described as patients rather than adults. Women were slightly more numerous than men (53.3% versus 46.7%), and the population was predominantly urban (77.9%), consistent with referral patterns to two tertiary centres in a regional capital. The histological distribution was dominated by superficial spreading melanoma (67.8%), followed by nodular (17.8%), lentigo maligna (3.6%), and acral lentiginous (2.5%) subtypes. More than half of tumours (52.3%) were diagnosed at stage 0–I. Notably, 24 patients (6.1%) developed two or more primary melanomas during follow-up. The median follow-up of 2.9 years was shorter than the mean of 4.7 years because of a right-skewed distribution dominated by recently diagnosed patients, a structural feature that requires cautious interpretation of late-event estimates. By the last documented follow-up, 125 patients (31.7%) had died. Comparisons between patients with and without a second primary cancer are presented in Table 2.

The most striking contrast was age: SPC patients were on average more than a decade older at melanoma diagnosis (64.2 versus 53.7 years, *p* < 0.001). SPC patients also had significantly longer follow-up (6.92 versus 4.13 years, *p* = 0.001). This difference is structurally related to the opportunity to survive long enough to be classified as having an SPC and therefore creates unequal time at risk; subsequent between-group comparisons should be interpreted as descriptive rather than as time-adjusted effects. Sex and residence did not differ significantly between groups (*p* = 0.132 and *p* = 0.450, respectively). The index melanoma was thinner in the SPC group (Breslow 1.97 versus 2.78 mm, *p* = 0.044) and showed a lower mitotic rate (3.09 versus 5.53 per mm^2^, *p* = 0.004). Mortality was numerically lower in the SPC group (24.1% versus 33.7%), though the difference did not reach significance in the unadjusted comparison (*p* = 0.107). Site-specific incidence densities of second primary cancers are summarised in Table 3.

Across 1848 person-years, 79 patients accrued 131 distinct second-primary events, yielding an observed hospital-cohort incidence density of 70.9 per 1000 person-years (95% CI 59.3–84.1). The composition of these events was dominated by cutaneous malignancies, which accounted for 99 events and an incidence of 53.6 per 1000 person-years (95% CI 43.5–65.2), roughly three-quarters of the total burden. Solid-organ malignancies formed the second-largest group at 14.6 per 1000 person-years (95% CI 9.6–21.3), showing that the observed SPC burden was not confined to the skin. Haematologic and endocrine second primaries were rare, at 1.6 and 1.1 per 1000 person-years respectively, with wide confidence intervals because of the small event counts. Median latency was similar across cutaneous (3.29 years), solid-organ (3.10 years), and endocrine (4.17 years) categories, whereas the three haematologic events occurred early (median 0.54 years), a pattern compatible with surveillance at melanoma work-up or synchronous detection. Because synchronous and metachronous SPCs were not formally separated, this interpretation remains descriptive. The Kruskal–Wallis comparison of latency across categories was H = 1.41 (*p* = 0.702). Constitutional and lifestyle factors by SPC status are compared in Table 4.

Among constitutional and lifestyle factors, a personal history of actinic keratosis emerged as by far the strongest correlate of second-primary development. Actinic keratosis was present in 36.8% of SPC patients but only 8.0% of those without an SPC, a highly significant difference (*p* < 0.001) corresponding to an unadjusted odds ratio of 6.64 (95% CI 3.42–12.92). The classical pigmentary risk factors for melanoma itself, namely fair phototype and red or blond hair, did not distinguish the two groups (*p* = 0.336 and *p* = 0.546), nor did a high naevus count (*p* = 0.297). Immunosuppression was numerically more frequent among SPC patients (2.5% versus 0.6%) but the small numbers precluded significance (*p* = 0.181), and lifestyle exposures including alcohol use and occupational noxious exposure showed no association (*p* = 0.892 and *p* = 0.263). Melanoma tumour characteristics and treatment by SPC status are summarised in Table 5.

Tumour-level characteristics largely failed to separate the two groups. Anatomical location showed a numerically higher proportion of head-and-neck melanomas among SPC patients (19.0% versus 10.8%), consistent with a possible role for chronic sun exposure, but the overall distribution of location did not reach significance. Clinicopathological subtype was similarly distributed, with superficial spreading melanoma predominating in both groups and the chi-square comparison non-significant (*p* = 0.107). Stage at diagnosis did not differ meaningfully (*p* = 0.599 for the overall distribution). Melanoma immunotherapy had been administered to 7.6% of SPC patients compared with 18.1% of SPC-absent patients (*p* = 0.025). Because immunotherapy is generally used in more advanced disease, treated patients may have had shorter time available to develop an SPC and a higher competing risk of melanoma-specific death. This descriptive association should not be interpreted as a protective treatment effect and requires confirmation with time-dependent treatment exposure and competing-risk methods. Sentinel-node positivity, available in a subset, showed no association (*p* = 0.390). The results of the multivariable logistic regression are presented in Table 6.

**Figure 1:**
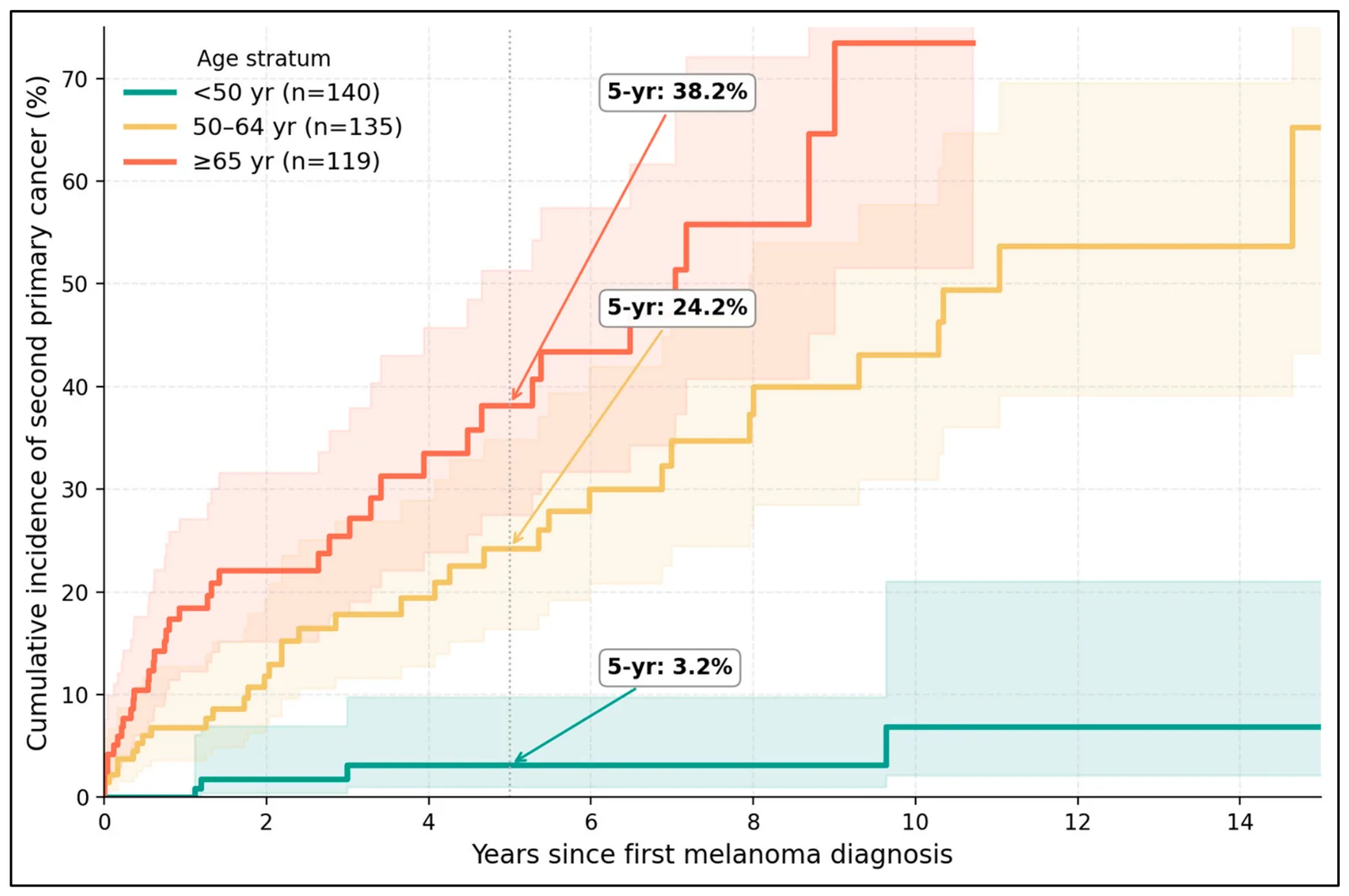
Cumulative incidence of a second primary cancer over time since first melanoma diagnosis, estimated as the complement of the Kaplan–Meier function and stratified by age at diagnosis (<50, 50–64, ≥65 years), with 95% confidence bands. By five years the cumulative incidence reaches 38.2% in patients aged 65 years or older, 24.2% in the 50–64 years stratum, and 3.2% in those under 50, as marked at the five-year reference line. Late curve tails beyond five years should be interpreted cautiously because the median follow-up was 2.9 years and death was censored rather than modelled as a competing event.

**Figure 2:**
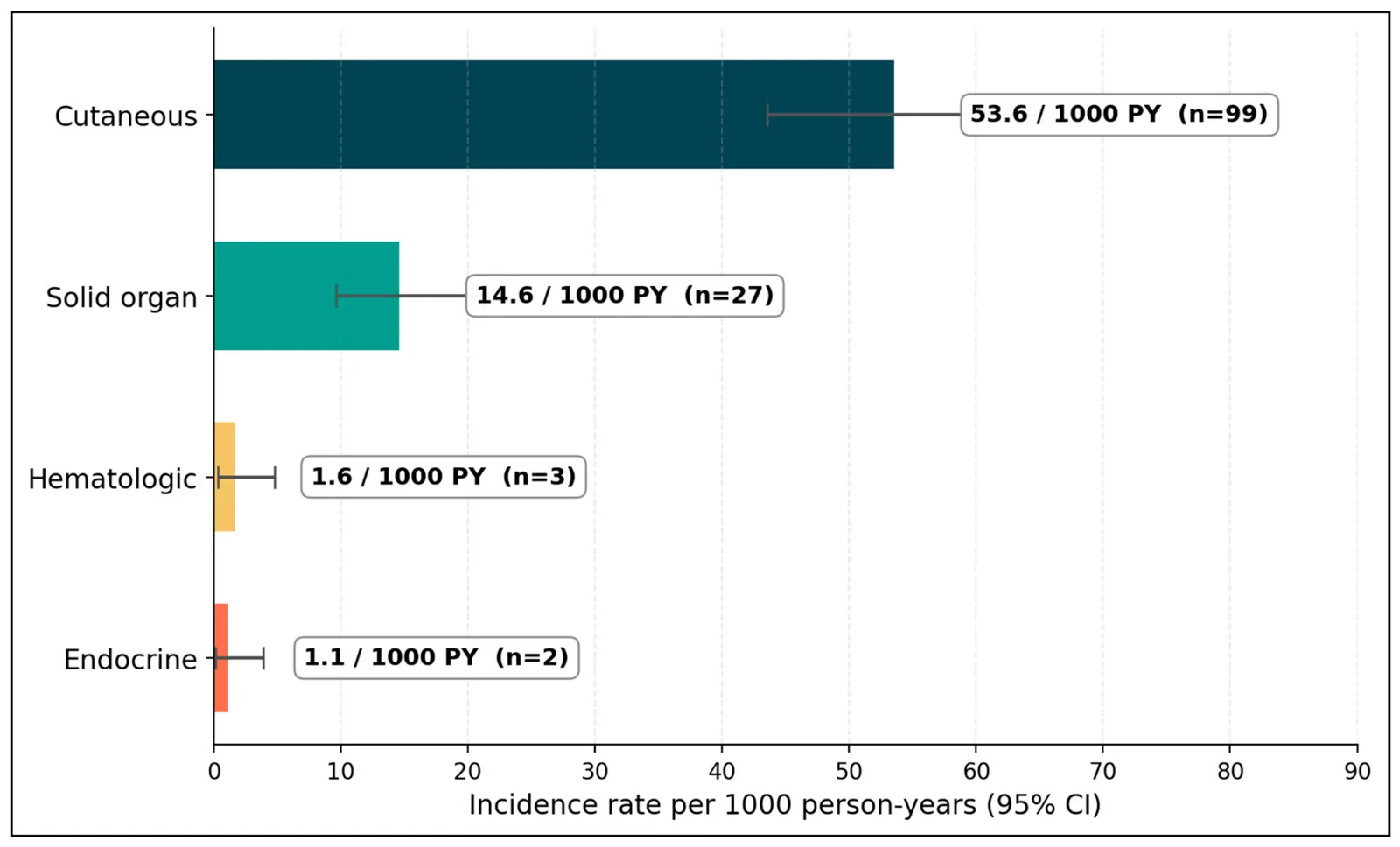
Site-specific incidence density of second primary cancers per 1000 person-years with Poisson 95% confidence intervals. Cutaneous second primaries dominate at 53.6 per 1000 person-years (99 events), followed by solid-organ (14.6 per 1000 person-years; 27 events), haematologic (1.6 per 1000 person-years; 3 events), and endocrine (1.1 per 1000 person-years; 2 events) malignancies.

**Figure 3:**
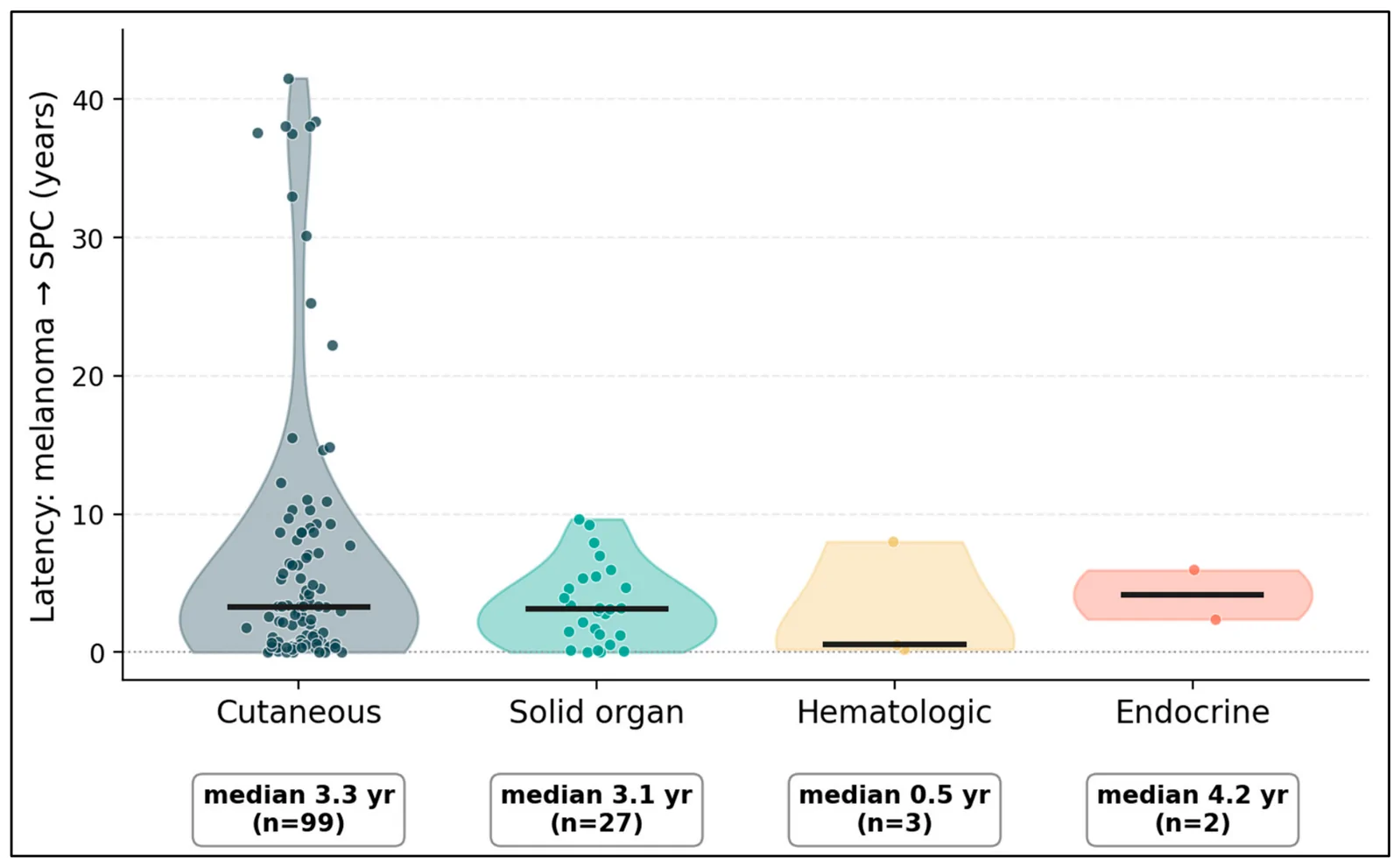
Distribution of latency from melanoma to each second primary, shown as violin plots overlaid with individual events and the group median. Median latency was 3.3 years for cutaneous, 3.1 years for solid-organ, and 4.2 years for endocrine second primaries, while the three haematologic events occurred earliest (median 0.5 years).

Age remained the dominant determinant: each additional decade of age at melanoma diagnosis raised the odds of a subsequent primary cancer by 86% (adjusted OR 1.86, 95% CI 1.49–2.32, *p* < 0.001). Neither male sex (OR 1.26, *p* = 0.415) nor urban residence (OR 0.78, *p* = 0.434) contributed independently once age was accounted for. The model also showed an inverse association with Breslow thickness: each additional millimetre of tumour depth was associated with lower odds of an observed SPC (adjusted OR 0.86, 95% CI 0.76–0.96, *p* = 0.008). This counterintuitive association should not be interpreted as protective and is considered likely to reflect differential survival and competing melanoma mortality, as discussed below. The age-stratified cumulative incidence of a second primary cancer is illustrated in Figure 1, the site-specific incidence densities are shown in Figure 2, and the latency distributions are shown in Figure 3. The exploratory Cox proportional-hazards model for overall survival is presented in Table 7.

The exploratory Cox model summarised the association between fixed SPC status and overall survival after adjustment for age, sex, and Breslow thickness. SPC status was associated with a lower observed hazard of death (HR 0.36, 95% CI 0.21–0.63, *p* < 0.001), and the log-rank comparison was concordant (*p* = 0.005), with median overall survival not reached in the SPC group versus 10.7 years in patients without an SPC. This should not be interpreted as a protective effect of developing another cancer. Each year of age increased the hazard by 4% (HR 1.04, *p* < 0.001) and each millimetre of Breslow thickness by 14% (HR 1.14, *p* < 0.001), while male sex showed a non-significant trend toward higher mortality (HR 1.29, *p* = 0.187). Because SPC status was modelled as a fixed exposure that can only occur after a period of survival, the SPC hazard ratio is subject to immortal-time and detection bias and should be regarded as preliminary until confirmed with time-varying or multi-state methods.

The Kaplan–Meier complement estimates rose from 7.7% at one year to 14.8% at three years and 20.7% at five years. At five years the estimate was 38.2% in patients aged 65 years or older, 24.2% in the 50–64 years stratum, and 3.2% in those under 50. The Cochran–Armitage trend test confirmed a monotonic association between age stratum and SPC occurrence (Z = 5.63, *p* < 0.001). Ten-year values are shown in Table 8 only as descriptive late-tail estimates; given the median follow-up of 2.9 years, sparse observation at later time points, and censoring of death rather than competing-risk estimation, they should not be interpreted as stable absolute risks. The multiplicity of second primaries and the prior-cancer subgroup are summarised in Table 9.

Among the 79 patients who developed a second primary, the majority (55) had a single SPC, but a substantial minority experienced recurrent multiple primaries, with 14 patients accruing two SPCs, 10 accruing three or more, and one exceptional patient accumulating eight distinct malignant events. A total of 31 patients (7.9%) had been diagnosed with a cancer before their melanoma, and these individuals were markedly older than the rest of the cohort at melanoma diagnosis (68.5 versus 54.8 years, *p* < 0.001), again pointing to age as the common thread linking malignant events across a lifetime. A history of prior cancer was associated with a non-significant increase in the odds of subsequently developing yet another primary after melanoma (OR 1.71, 95% CI 0.76–3.88, *p* = 0.240).

## 4. Discussion

This retrospective hospital-based cohort found that 79 of 394 patients developed at least one SPC and that the observed event incidence density was 70.9 per 1000 person-years. These absolute estimates should not be interpreted as population-relative “excess risk” because the study did not include a matched general-population comparator. International studies remain useful for context: the meta-analysis by Caini and colleagues reported a relative risk of approximately 1.6 for non-melanoma second primaries [4], while population-based cohorts from Spain and Korea reported cumulative probabilities or standardised incidence ratios [8,9]. Those measures are methodologically different from the crude hospital-cohort incidence reported here and are not directly interchangeable. The higher observed proportion in our series may also be influenced by referral intensity and the inclusion of keratinocyte carcinomas, which some registries omit.

The predominance of cutaneous SPCs is clinically important and is consistent with shared ultraviolet exposure. Melanoma survivors are known to carry increased risks of basal-cell and squamous-cell carcinoma [10], and cumulative sun exposure contributes substantially to melanoma burden [1,2]. In our cohort, actinic keratosis was the strongest unadjusted correlate of SPC occurrence, with an unadjusted odds ratio of 6.64; because it was not included in the multivariable model, it should not be described as an independent predictor. Actinic keratosis remains a plausible marker of chronic photodamage and field cancerization [16]. However, the present data do not establish that photodamage predominates over constitutional susceptibility. Naevus burden, inherited predisposition, and other genetic factors were not comprehensively measured, and prior work indicates that these factors can be important for multiple primary melanomas [13]. The non-cutaneous SPCs observed in this cohort, principally solid-organ tumours, are also consistent with the broader range of malignancies reported among melanoma and other cancer survivors [6,24,25,26,27].

A central methodological issue is that several apparently favourable associations are readily explained by time-at-risk and competing mortality. The most likely explanation for the inverse association between Breslow thickness and observed SPC occurrence is competing melanoma mortality: patients with thicker, more aggressive melanomas are more likely to die earlier and therefore have less opportunity to survive long enough for a new primary to be diagnosed. The same structure contributes to the apparently protective survival association of SPC status and to the lower frequency of immunotherapy among SPC patients. Fixed classification of patients according to whether an SPC ever occurred creates immortal time before the SPC diagnosis [23]. Consequently, the conventional Cox estimate reported here is preliminary and should not be interpreted as a causal survival effect. Time-varying exposure models, multi-state approaches, target-trial principles, and competing-risk methods are better suited to definitive analyses [28].

The age gradient and the association with markers of chronic photodamage may help generate hypotheses for risk-stratified survivorship care, but this retrospective cohort does not establish the safety of reducing standard surveillance for younger patients or those with thin melanomas. Previous studies demonstrate that second-cancer risk varies by age and melanoma subtype [12,29], but they do not by themselves validate de-escalation of follow-up. Any change in surveillance intensity should therefore be tested in prospective, externally validated risk models. Patients with multiple primary melanomas remain an important group for continued dermatologic surveillance [5]. Immunosuppression was uncommon in this cohort and was not significantly associated with SPC occurrence (2.5% versus 0.6%, *p* = 0.181); thus, no cohort-specific inference can be made for this subgroup. The clinical relevance of immunosuppression is discussed only in light of external evidence showing increased and sometimes more aggressive cutaneous malignancy in transplant recipients and other immunosuppressed populations [14,15,18], rather than as a finding demonstrated by the present analysis.

Finally, the Romanian and wider Eastern European context underscores the need for locally validated data. Geographic differences in melanoma incidence, mortality, surveillance resources, and cancer ascertainment can affect observed SPC patterns [3]. The present study provides an initial hospital-based description from two referral centres, but it should be viewed as hypothesis-generating rather than population-representative. Linking institutional cohorts to national cancer and mortality registries would allow standardised incidence ratios against the Romanian population, more complete ascertainment of cancers diagnosed outside the referral centres, and longer follow-up for late solid-organ SPCs.

## 5. Study Limitations

Several limitations temper these findings. First, the retrospective design depended on the completeness and accuracy of existing clinical records and may have introduced information and misclassification bias. Second, the cohort was hospital-based and drawn from only two tertiary referral centres, creating referral, selection, and surveillance bias and limiting the generalisability of absolute incidence estimates to the wider Romanian population. Third, follow-up was relatively short and right-skewed, with a median of 2.9 years. The late tails of the Kaplan–Meier complement curves are therefore unstable; exact numbers remaining at risk at later time points could not be reconstructed from the aggregate analytic output available for this study, and ten-year estimates should be considered descriptive only. Fourth, death was censored rather than handled as a competing event for SPC incidence, so Kaplan–Meier complement estimates may overstate absolute risk. Fifth, fixed SPC status in the survival model creates immortal-time and detection bias; the observed lower mortality among SPC patients is preliminary and should not be interpreted as protective. Sixth, the absence of a matched general-population reference precluded standardised incidence ratios. Seventh, the adjusted predictor model used 381 complete cases, excluding 13 patients (3.3%) with missing model covariates. The source analysis file did not preserve a screening log of records excluded before final cohort assembly, a single administrative recruitment-window field, or a separate loss-to-follow-up indicator beyond last documented contact; it also lacked sufficient metadata to perform a sensitivity analysis of the excluded records without returning to source records. Finally, some risk-factor fields were incomplete, synchronous and metachronous SPCs were not formally separated, constitutional/genetic susceptibility was not comprehensively measured, and the small numbers of haematologic, endocrine, prior-cancer, and immunosuppressed cases limited subgroup inference.

## 6. Future Directions

Future studies should validate these findings in larger multicentre and population-based Romanian cohorts linked to national cancer and mortality registries, with prospectively defined recruitment windows, screening logs, loss-to-follow-up reporting, and standardised ascertainment of new primary cancers. Analyses should distinguish synchronous from metachronous SPCs using a prespecified interval and should model repeated SPCs with recurrent-event or multi-state approaches. Death should be treated as a competing event using cumulative-incidence functions and cause-specific or Fine–Gray models, while SPC status and treatment exposures such as immunotherapy should be handled as time-varying variables in survival analyses. External validation should examine whether age, actinic damage, melanoma characteristics, ultraviolet exposure, naevus burden, germline susceptibility, and immune status can be combined into reproducible risk models. Comparisons with the general Romanian population should use standardised incidence ratios or matched population controls. Most importantly, surveillance strategies suggested by retrospective risk gradients should be tested prospectively for clinical benefit, resource use, and safety before either intensification or de-escalation is recommended for specific patient groups.

## 7. Conclusions

Melanoma survivors in this two-centre hospital cohort experienced a clinically relevant burden of additional primary cancers, predominantly cutaneous malignancies. Older age independently predicted observed SPC occurrence, while actinic keratosis was a strong unadjusted marker consistent with chronic photodamage. The inverse association with Breslow thickness and the apparent survival advantage among SPC patients are likely influenced by competing mortality, unequal time at risk, detection bias, and immortal-time bias. The findings should therefore be considered hypothesis-generating and require population-based external validation with competing-risk and time-dependent methods before they are used to individualise surveillance intensity.

## Figures and Tables

**Table 1:** Baseline characteristics of the melanoma cohort (*N* = 394).

Characteristic	Value
Patients, *n*	394
Age at first melanoma, mean ± SD, yr	55.8 ± 14.7
Age range, yr	16.3–91.4
Female sex, *n* (%)	210 (53.3)
Male sex, *n* (%)	184 (46.7)
Urban residence, *n* (%)	307 (77.9)
Rural residence, *n* (%)	87 (22.1)
Total follow-up, person-years	1848
Median follow-up, yr	2.9
Superficial spreading melanoma, *n* (%)	267 (67.8)
Nodular melanoma, *n* (%)	70 (17.8)
Lentigo maligna melanoma, *n* (%)	14 (3.6)
Acral lentiginous melanoma, *n* (%)	10 (2.5)
Stage 0–I at diagnosis, *n* (%)	206 (52.3)
Patients with ≥2 primary melanomas, *n* (%)	24 (6.1)
Deceased at last follow-up, *n* (%)	125 (31.7)

**Table 2:** Comparison of patients with versus without a second primary cancer.

Variable	SPC Present (*n* = 79)	SPC Absent (*n* = 315)	*p*-Value	Test
Age, mean ± SD, yr	64.2 ± 12.7	53.7 ± 14.4	<0.001	Welch t
Follow-up, mean ± SD, yr	6.92 ± 6.98	4.13 ± 4.05	0.001	Welch t
Male sex, *n* (%)	43 (54.4)	141 (44.8)	0.132	Fisher
Urban residence, *n* (%)	59 (74.7)	248 (78.7)	0.450	Fisher
Breslow, mean ± SD, mm	1.97 ± 2.80	2.78 ± 4.04	0.044	Welch t
Mitotic rate, mean ± SD/mm^2^	3.09 ± 4.57	5.53 ± 11.40	0.004	Welch t
Deceased, *n* (%)	19 (24.1)	106 (33.7)	0.107	Fisher

**Table 3:** Site-specific incidence density of second primary cancers.

SPC Category	Events, *n*	Incidence/1000 PY	95% CI	Median Latency, yr
Cutaneous malignancy	99	53.6	43.5–65.2	3.29
Solid-organ malignancy	27	14.6	9.6–21.3	3.10
Haematologic malignancy	3	1.6	0.3–4.7	0.54
Endocrine malignancy	2	1.1	0.1–3.9	4.17
All SPC events	131	70.9	59.3–84.1	3.20

**Table 4:** Constitutional and lifestyle risk factors associated with second primary cancer.

Risk Factor	SPC Present, *n*/*N* (%)	SPC Absent, *n*/*N* (%)	*p*-Value	Test
History of actinic keratosis	25/68 (36.8)	21/261 (8.0)	<0.001	Fisher
Fair phototype (I–II)	14/60 (23.3)	63/210 (30.0)	0.336	Fisher
Red or blond hair	—	—	0.546	Fisher
High naevus count (>50)	18/64 (28.1)	82/229 (35.8)	0.297	Fisher
Immunosuppression	2/79 (2.5)	2/315 (0.6)	0.181	Fisher
Alcohol use	—	—	0.892	Fisher
Occupational noxious exposure	—	—	0.263	Fisher

**Table 5:** Melanoma tumour characteristics and treatment by SPC status.

Variable	SPC Present (*n* = 79)	SPC Absent (*n* = 315)	*p*-Value	Test
Head/neck location, *n* (%)	15 (19.0)	34 (10.8)	—	χ^2^
Trunk location, *n* (%)	37 (46.8)	150 (47.6)	—	χ^2^
Limb location, *n* (%)	25 (31.6)	114 (36.2)	0.21	χ^2^ (overall)
SSM subtype, *n* (%)	56 (70.9)	211 (67.0)	0.107	χ^2^ (overall)
Stage 0–I, *n* (%)	47 (59.5)	159 (50.5)	0.599	χ^2^ (overall)
Stage III–IV, *n* (%)	13 (16.5)	64 (20.3)	—	—
Melanoma immunotherapy, *n* (%)	6 (7.6)	57 (18.1)	0.025	Fisher
SLNB positive, *n* (%)	—	—	0.390	Fisher

**Table 6:** Multivariable logistic regression for second primary cancer (*n* = 381).

Predictor	Adjusted OR	95% CI	*p*-Value
Age, per 10-year increase	1.86	1.49–2.32	<0.001
Male sex	1.26	0.73–2.17	0.415
Urban residence	0.78	0.41–1.46	0.434
Breslow thickness, per mm	0.86	0.76–0.96	0.008

**Table 7:** Cox proportional-hazards model for overall survival (*n* = 381).

Covariate	Hazard Ratio	95% CI	*p*-Value
Second primary cancer (yes vs. no)	0.36	0.21–0.63	<0.001
Age, per year	1.04	1.03–1.06	<0.001
Male sex	1.29	0.88–1.87	0.187
Breslow thickness, per mm	1.14	1.11–1.18	<0.001

**Table 8:** Kaplan–Meier complement estimates of first second primary cancer occurrence and age trend.

Time Horizon	Cumulative SPC Incidence (%)	Age < 50 (%)	Age 50–64 (%)	Age ≥ 65 (%)
1 year	7.7	—	—	~22
3 years	14.8	~2	~20	~30
5 years	20.7	3.2	24.2	38.2
10 years	36.2	~7	~54	~73
Cochran–Armitage trend	—	Z = 5.63	—	*p* < 0.001

**Table 9:** Multiplicity of second primaries and the prior-cancer subgroup.

Metric	Value
Patients with exactly 1 SPC, *n*	55
Patients with 2 SPCs, *n*	14
Patients with ≥3 SPCs, *n*	10
Maximum SPCs in one patient, *n*	8
Patients with a prior (pre-melanoma) cancer, *n* (%)	31 (7.9)
Prior cancer & subsequent SPC, *n*	9
Prior cancer vs. SPC association, OR (95% CI)	1.71 (0.76–3.88)
Prior cancer vs. SPC, *p*-value (Fisher)	0.240
Mean age, prior-cancer vs. none, yr	68.5 vs. 54.8 (*p* < 0.001)

## Data Availability

The data presented in this study are available on request from the corresponding author. The data are not publicly available due to privacy and ethical restrictions.
